# Hypercapnia at admission, regardless of acidosis, may worsen the outcome of hospitalised patients with chronic obstructive pulmonary disease exacerbations

**DOI:** 10.1007/s11739-026-04279-0

**Published:** 2026-02-09

**Authors:** Giulia Sartori, Alberto Fantin, Filippo Sartori, Albert Gabarrús, Ernesto Crisafulli, Antoni Torres

**Affiliations:** 1 Department of Medicine, Respiratory Medicine Unit, University of Verona and Azienda Ospedaliera Universitaria Integrata of Verona, Verona, Italy; 2 Department of Pulmonology, S. Maria Della Misericordia University Hospital, Udine, Italy; 3 Department of Pulmonology, Clinic Institute of Thorax (ICT), Hospital Clinic of Barcelona—Institut d’Investigacions Biomèdiques August Pi i Sunyer (IDIBAPS), University of Barcelona—Ciber de Enfermedades Respiratorias (CIBERES), Villarroel 170, 08036 Barcelona, Spain

**Keywords:** COPD exacerbations, Hospitalisation, Hypercapnia, Acidosis, Prognosis, Outcomes

## Abstract

In patients with exacerbation of chronic obstructive pulmonary disease (ECOPD), the presence of respiratory acidosis is considered “*a marker of severity*”, identifying the need for ventilation treatment. The evaluation of ECOPD patients with hypercapnia, even without acidosis, may provide information on patients not commonly defined as “*at risk*”. We retrospectively assessed 407 hospitalised patients with ECOPD, divided into three groups at admission: patients with normocapnia (*N* = 176), hypercapnia (PaCO_2_ > 45 mmHg and pH ≥ 7.35, *N* = 126), and acidosis (PaCO_2_ > 45 mmHg and pH < 7.35, *N* = 105). Data on general, clinical, laboratory and microbiological characteristics, and on outcomes (mortality up to 3 years), were collected. Patients with hypercapnia and acidosis had similar functional and clinical characteristics to their normocapnic peers, but of greater severity. The mortality rate at 1 year was similar between hypercapnic and acidotic patients (24% and 25%), and higher than that recorded in the normocapnic group (14%). Similar trends were observed in mortality from 6 months to 3 years. The presence of hypercapnia and acidosis was significantly associated with an increased and independent risk of death at 1 year. A threshold value of PaCO_2_ ≥ 55 mmHg was associated with 1-year mortality. In a multivariate analysis considering the new threshold and compared to normocapnia, the presence of hypercapnia (PaCO_2_ ≥ 55), regardless of acidosis, was independently associated with a higher mortality risk at 1 year. In hospitalised ECOPD patients, hypercapnia itself, even when compensated, is associated with a worse prognosis up to an intermediate-term follow-up. The need to ventilate these patients should be evaluated, regardless of the presence or absence of acidosis.

**Supplementary Information:**

The online version contains supplementary material available at 10.1007/s11739-026-04279-0.

## Introduction

Chronic obstructive pulmonary disease (COPD) represents a significant and increasing challenge for health systems worldwide, due to its high impact on morbidity, disability, and mortality [1]. Multiple factors contribute to progressive skeletal muscle wasting in COPD patients [2], and in those who also have severe airway obstruction, the presence of alveolar hypoventilation may impair gas exchange, leading to hypercapnia [3]. The presence of hypercapnia, defined by an increase in the partial pressure of arterial carbon dioxide (PaCO_2_) >45 mmHg, is therefore a sign of ventilatory exhaustion and has not only pulmonary but also systemic impacts, including cardiovascular and musculoskeletal consequences [3]. In very severe COPD patients, the presence of hypercapnia is around 25% [4].

The exacerbation of COPD (ECOPD), interrupting the stable phase of the disease, worsens several outcomes, especially if patients need to be hospitalised [5]. In these patients, hypercapnia is primarily caused by mechanical alterations in the ventilation/perfusion (*V*/*Q*) ratio, resulting from airway resistance (bronchospasm), flow limitation, oedema of the airway wall and accumulation of mucus hypersecretion [3]. Moreover, the hyperactivity of the respiratory neural drive, characterised by a rapid and shallow breathing pattern, causes dynamic hyperinflation and pulmonary entrapment and an increase in the intrinsic positive end-expiratory pressure (iPEEP), which represents an additional mechanical load (work of breathing, WOB) which the respiratory system must overcome in order to guarantee respiratory gas exchanges [3]. All these factors, leading to increased oxygen demand and a reduction in muscle respiratory efficiency, may contribute to the development of hypercapnia during ECOPD [3, 6]. The presence of hypercapnia may be associated with respiratory acidosis, defined by a pH < 7.35 [6]. At admission due to ECOPD, the presence of acidosis is around 20% [7].

Bilevel non-invasive mechanical ventilation (NIMV), a key treatment for addressing iPEEP and WOB in ECOPD patients with hypercapnia and acidosis, was proposed in the 2017 European Respiratory Society (ERS) guidelines with strong evidence-based recommendations [8]. However, the same guidelines conditionally recommend that NIMV not be used in ECOPD patients with hypercapnia without acidosis [8]. In 2019, however, the ERS task force conditionally supported the long-term domiciliary use of NIMV in stable COPD with chronic hypercapnia to improve health outcomes [9].

In the recent definition and grading of ECOPD (the Rome proposal), the presence of acidosis was considered a marker of ECOPD, indicating severity, which differentiates it from moderate (characterised by hypercapnia without acidosis) and mild grades (considered the default) [10]. In terms of prognosis, however, the Rome classification may not be able to discriminate between patients with moderate or severe disease [11]. These findings brought into question whether hypercapnia without acidosis can be harmful to patients with ECOPD. The hypothesis of this study is that hypercapnia may play a predominant role in patients admitted for ECOPD. Therefore, the study aimed to evaluate the impact of hypercapnia at admission and the outcomes of hospitalised patients with ECOPD.

## Methods

### Study design and patients

We retrospectively analysed data of prospectively hospitalised ECOPD patients. The sampling method was systematic, and all patients with ECOPD admitted to the Pulmonology Department of Hospital Clínic of Barcelona (Spain) between May 2009 and March 2018 were considered for enrolment. COPD was confirmed by clinical history and spirometry performed during a stable phase at least 6 months prior to admission, when fixed airflow obstruction was evaluated after administration of a bronchodilator (salbutamol). COPD and ECOPD were defined according to the GOLD 2023 document [12], and the need for hospitalisation was established based on ECOPD severity in accordance with clinical signs and symptoms and the presence of potential indicators [13]. Acute respiratory failure was defined according to the values of arterial blood gas (ABG) measured at admission: the ratio of partial pressure of arterial oxygen to the fraction of inspired oxygen (PaO_2_/FiO_2_) <300 defined hypoxemia, while hypercapnia was defined when patients had a PaCO_2_ > 45 mmHg, with or without acidosis (pH < 7.35 and pH ≥ 7.35, respectively) [8]. Among admitted patients, we excluded those with concomitant chronic respiratory disease other than COPD (such as asthma or pulmonary fibrosis), as well as patients with community-acquired pneumonia (CAP) or acute heart failure, identified by clinical and radiological criteria. A consolidation on the thorax X-ray, accompanied by a higher inflammatory response as indicated by C-reactive protein (CRP) >12.9 mg/dL, was used as a criterion to identify CAP in patients with COPD and to differentiate it from ECOPD [14]. Moreover, we excluded patients in whom an ABG was not performed and those with metabolic acidosis (pH < 7.35 and PaCO_2_ ≤ 45 mmHg). The hospital’s Ethics Committee approved the study protocol (CEIC 2008/4106), and the study was conducted in accordance with good clinical practices and the Declaration of Helsinki. All enrolled patients provided informed consent.

### Definition of study groups

The study cohort was divided into three groups: patients with normocapnia (PaCO_2_ ≤ 45 mmHg), patients with compensated hypercapnia (pH ≥ 7.35 and PaCO_2_ > 45 mmHg, hence defined only as hypercapnia), and patients with respiratory acidosis (pH < 7.35 and PaCO_2_ > 45 mmHg, hence defined only as acidosis). Supplementary Fig. 1 displays a flow diagram of the study.

### Measurements

We considered data on demographic variables, body mass index (BMI), smoking habit (never, current or former), with the number of packs/years, number of co-morbidities (Charlson index), dyspnoea grade measured by the modified Medical Research Council (mMRC) scale, and the use of long-term oxygen therapy (LTOT). The number of exacerbations occurring in the previous year was also recorded. Furthermore, respiratory rate and heart rate were assessed at admission. In addition to ABG data (pH, PaO_2_/FiO_2_, PaCO_2_, HCO_3_^−^-serum bicarbonate, and BE-base excess), other laboratory values (leucocytes, haemoglobin, CRP and creatinine) were recorded at admission and day 3. Moreover, the number of patients using systemic corticosteroids and antibiotics was recorded, as well as the number of positive sputum cultures, with the prevalence of microbiologic agents.

### Primary outcome

The primary outcome was all-cause, 1-year mortality (since admission).

### Secondary outcomes

Secondary outcomes were: (a) all-cause, in-hospital and at 30- and 90-day, 6-, 24- and 36-month mortality (since admission); (b) length of hospital stay (LHS); (c) the use of NIMV or invasive mechanical ventilation (IMV); (d) the need for intensive care unit (ICU) admission; (e) occurrence of new episodes of ECOPD and related readmissions within 30 days of discharge.

### Statistical analysis

For categorical variables, we reported the number and percentage of patients by category, and for continuous variables the median (IQR-interquartile range). To compare categorical and continuous variables among study groups, we used the chi-squared test or the Freeman-Halton extension [15] of the Fisher exact test for contingency tables that were larger than 2 × 2, when appropriate, and the non-parametric Kruskal–Wallis test, respectively. We performed pairwise comparisons to compare different study groups using the Bonferroni method only for secondary outcomes. The assumption of normality was checked with Kolmogorov–Smirnov tests.

Survival curves for the study groups were obtained using the Kaplan–Meier method and compared using the Gehan–Breslow–Wilcoxon test [16]. We then analysed the association between the study groups and 1-year mortality using a Cox regression multivariable model [17] adjusting for potential confounders. The following variables, selected according to their potential clinical relevance, were included in the analysis: age, sex, body mass index (BMI), smoking habit, forced expiratory volume in the first second (FEV_1_), LTOT, Charlson Index and previous ECOPD. Regarding exacerbations, we retained the variable “number of previous ECOPD ≥ 2” for the multivariable analysis, while “previous hospitalisation/s for ECOPD” was excluded due to high collinearity with the former. Single collinearity in the multivariable analysis was evaluated using the Spearman correlation coefficient (*r*); multicollinearity was examined using the variance inflation factor (VIF), and variables with high collinearity (*r* >|± 0.50|) were excluded. Proportional hazard assumptions were tested using log-minus-log plots. Hazard ratios (HRs) and their 95% confidence intervals (CIs) were calculated. Assessment of discrimination in the multivariable model was measured using Harrell’s C and Somers’ D. Patients who were lost to follow-up were censored in survival analyses. We also performed appropriately adjusted analyses for secondary endpoints. Finally, the receiver operating characteristic (ROC) analysis, with the area under the curve (AUC), was used to identify the threshold of PaCO_2_ which was significantly stratified with the mortality at 1 year. A multivariable Cox regression model was repeated with the threshold of PaCO_2_ in order to assess factors associated with mortality at 1 year.

The level of significance was set at 0.05 (two-tailed). All analyses were performed using IBM SPSS version 26.0 (IBM Corp., Armonk, NY, USA).

## Results

### General, clinical, laboratory and microbiological results

We considered 407 patients with ECOPD, divided into three groups: patients with normocapnia (*N* = 176, 43%), hypercapnia (*N* = 126, 31%), and acidosis (*N* = 105, 26%). Patients with normocapnia showed significant differences in smoking (number of packs/year), FEV_1_ and FEV_1_/FVC, as well as LTOT, with respect to patients with hypercapnia and acidosis. Patients with acidosis had a higher prevalence of comorbidities compared to those with normocapnia. Table 1 displays the general characteristics.

**Table 1:** General characteristics of study groups

Variables	Total cohort (*N* = 407)	Patients with normocapnia (*N* = 176)	Patients with hypercapnia (*N* = 126)	Patients with acidosis (*N* = 105)	*p*-value
Age, years	72 [14]	73 [15]	72 [12]	71 [13]	0.611
Male	323 (80)	135 (77)	97 (77)	91 (87)	0.111
BMI, kg/m^2^	27.4 [6.7]	27.3 [6.3]	27.5 [6.6]	27.5 [7.7]	0.928
Smoking habit					0.914
Never	14 (3)	5 (3)	5 (4)	4 (4)	
Current	163 (40)	70 (40)	48 (38)	45 (43)	
Former	230 (57)	101 (57)	73 (58)	56 (53)	
Packs/year	56 [40]	50 [30.5]	57.5 [50]	60 [40]	**0.012**^**a,c**^
FEV_1_, % of predicted	40.5 [24]	50 [28]	36 [22]	38 [14.5]	** <0.001**^**b,d**^
FEV_1_/FVC	47 [23]	52 [23.5]	47 [23]	43 [19.5]	**0.001**^**a,d**^
mMRC ≥2	172 (61)	66 (52)	66 (72)	40 (63)	0.742
GOLD 2023 stages					0.970
A	73 (23)	38 (27)	19 (18)	16 (22)	
B	98 (30)	42 (30)	34 (32)	22 (30)	
E	152 (47)	62 (44)	54 (50)	36 (49)	
Charlson index ≥2	232 (59)	86 (51)	77 (63)	69 (68)	**0.015**^**c**^
Ischaemic heart disease	38 (9)	19 (11)	9 (7.2)	10 (9.5)	0.563
Congestive heart disease	61 (15)	21 (12)	22 (18)	18 (17)	0.333
Diabetes	88 (22)	31 (18)	31 (25)	26 (25)	0.244
ECOPD ≥2^e^	96 (24)	40 (23)	34 (27)	22 (21)	0.588
ECOPD hospitalisations ≥1^e^	131 (32)	51 (29)	49 (39)	31 (30)	0.182
Domiciliary triple therapy	128 (37)	60 (39)	34 (32)	34 (39)	0.466
LTOT	111 (27)	29 (16)	45 (36)	37 (35)	** <0.001**^**a,c**^

Data are presented as the number of patients (percentage) or medians [interquartile range]. Percentages are calculated for non-missing data. In bold significant values.

^a^ and ^b^*p* < 0.05 and *p* < 0.001 for comparison between patients with normocapnia and hypercapnia, respectively;

^c^ and ^d^*p* < 0.05 and *p* < 0.001 for comparison between patients with normocapnia and acidosis, respectively;

^e^ occurring in the preceding year.

*BMI* indicates body mass index, *FEV*_1_ Forced expiratory volume in the 1 st second, *FVC* Forced vital capacity, *mMRC* Modified Medical Research Council, *GOLD* Global Initiative for Chronic Obstructive Lung Disease, *ECOPD* Exacerbation of the chronic obstructive pulmonary disease, *LTOT* Long-term oxygen therapy.

In the clinical and laboratory variables evaluated at admission (Supplementary Table 1), ABG measures differed among the three groups in pH, PaCO_2_, and BE, while PaO_2_/FiO_2_ and HCO_3_^−^ differed between patients with normocapnia and those with hypercapnia and acidosis. Again, at admission, CRP levels differed between hypercapnic patients (higher) and acidotic patients (lower), while the creatinine value was higher in patients with acidosis than in those with normocapnia and hypercapnia. At day 3, pH, PaCO_2_, HCO_3_^−^ and BE differed between patients with normocapnia and those with hypercapnia and acidosis. The prevalence of patients treated with systemic corticosteroids was higher in hypercapnic and acidotic groups than in the normocapnic group.

Among microbiological variables (Supplementary Table 2), the prevalence of patients having *Pseudomonas aeruginosa* infection was higher in the hypercapnia group, while that of *Streptococcus pneumoniae* was higher in the normocapnia group.

### Outcomes

#### Primary

**Fig. 1:**
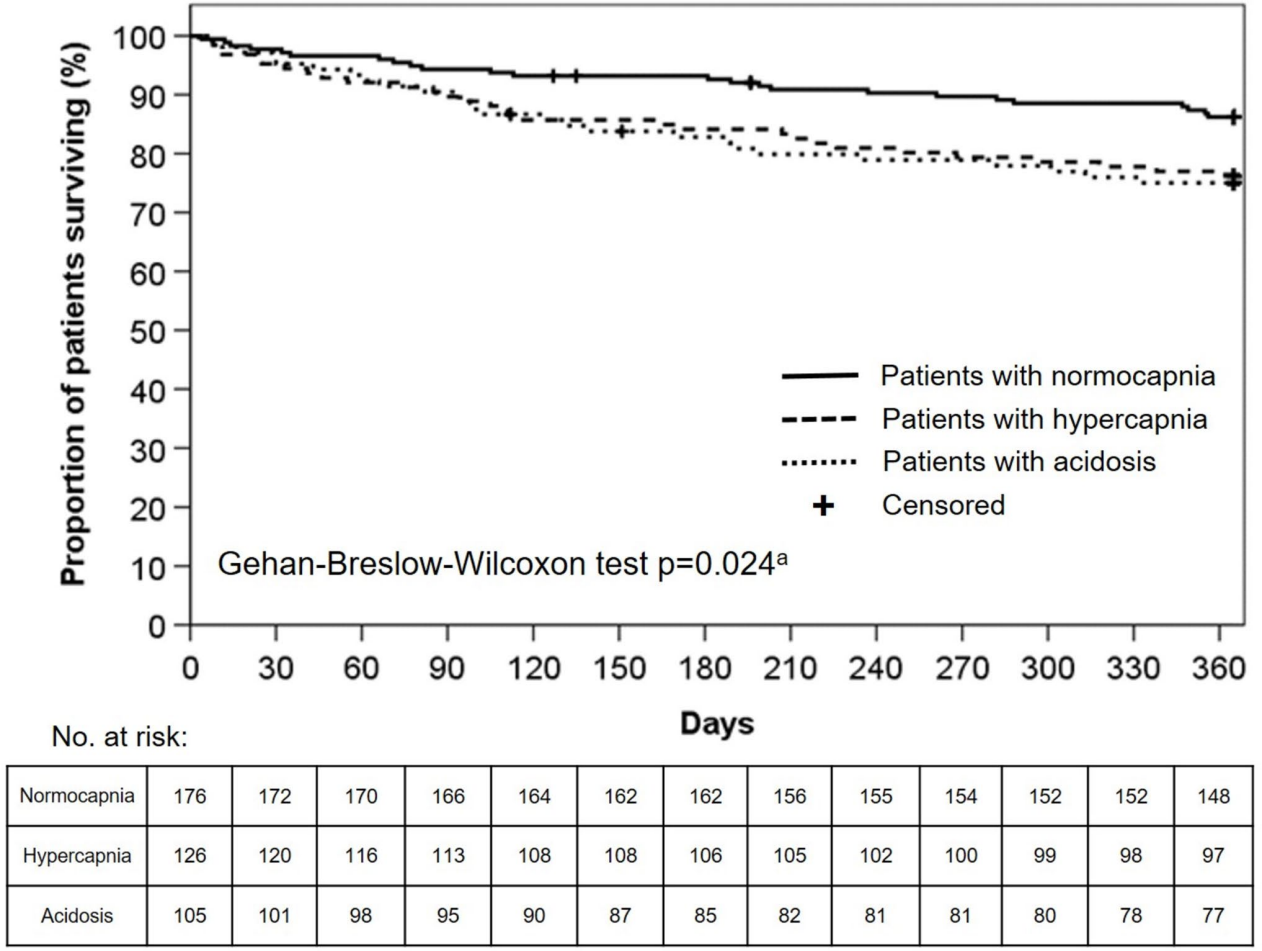
Kaplan–Meier survival curves at 1 year. ^a^*p* = 0.063 for comparison between patients with normocapnia and hypercapnia; *p* = 0.040 for comparison between patients with normocapnia and acidosis; *p* > 0.999 for comparison between patients with hypercapnia and acidosis

The all-cause mortality rate at 1 year was higher in patients with hypercapnia (24%) and acidosis (25%) than in those with normocapnia (14%), as well as the survival time. The Kaplan–Meier curves (Fig. 1) showed significant differences between the three study groups (Gehan–Breslow–Wilcoxon test, *p* = 0.024), particularly between patients with normocapnia and those with acidosis.

**Fig. 2:**
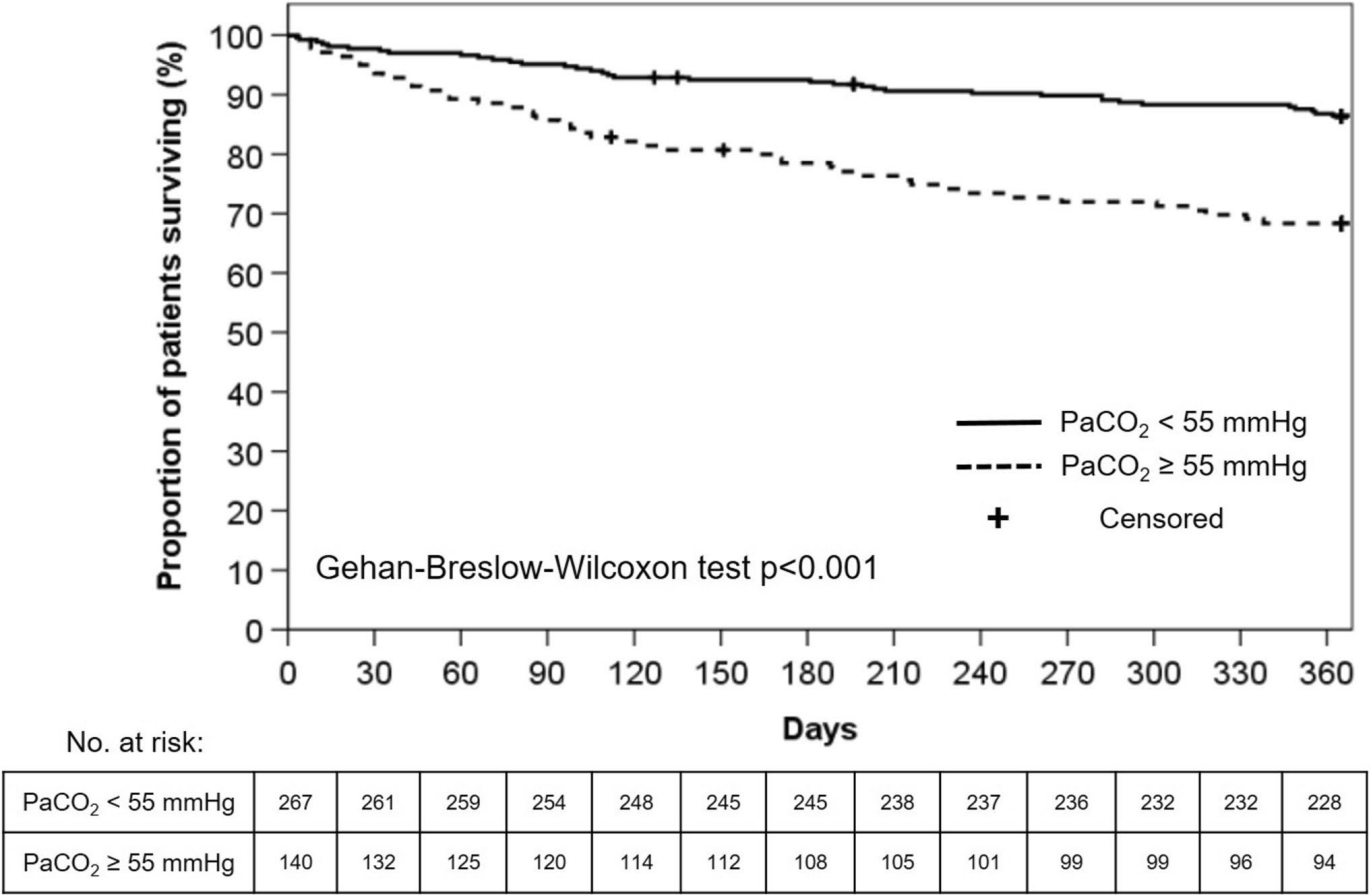
Kaplan–Meier survival curves at 1 year using the PCO_2_ cut-off of 55 mmHg

The ROC curve of PaCO_2_ (Supplementary Fig. 2) shows the best threshold of 55 mmHg associated with mortality at 1 year (AUC 0.625; SE 0.036; 95%CI 0.553 to 0.696; *p* = 0.001). The Kaplan–Meier curves at 1 year (Fig. 2) showed a significant difference between patients with a PaCO_2_ value of 55 mmHg or more and those with a value less than 55 mmHg (*p* < 0.001 on the Gehan–Breslow–Wilcoxon test).

#### Secondary

Differences in mortality were observed between patients with normocapnia (7.5%) and those with acidosis (17%) (at 6 months), between normocapnic (21%) and both hypercapnic and acidosis groups (34% and 37%) (at 2 years), and between normocapnic and hypercapnic (31% vs 46%) (at 3 years).

The LHS was shorter in patients with normocapnia (median, 7 days) than in those with hypercapnia and acidosis (8 and 9 days, respectively). The prevalence of patients treated with NIMV and IMV was significantly higher in acidosis than in the other two groups. The prevalence of patients admitted to the ICU showed a significant sequential increase between normocapnic, hypercapnic and acidosis, while the prevalence of patients having a new ECOPD within 30 days of discharge was higher in acidotic patients than in their normocapnic peers.

All study outcomes are reported in Table 2.

**Table 2:** Study outcomes

Variables	Patients with normocapnia (*N* = 176)	Patients with hypercapnia (*N* = 126)	Patients with acidosis (*N* = 105)	*p*-value
Primary outcome				
Mortality at 1 year	24 (14)	30 (24)	26 (25)	**0.032**
Survival time, days	337 [325 to 349]	310 [291 to 330]	307 [285 to 328]	**0.024**^**c**^
Secondary outcomes				
In-hospital mortality	5 (2.8)	5 (4)	4 (3.8)	0.832
Mortality at 30 days	4 (2.3)	6 (4.8)	5 (4.8)	0.372
Mortality at 90 days	10 (5.7)	13 (10)	10 (9.5)	0.287
Mortality at 6 months	13 (7.5)	20 (16)	18 (17)	**0.023**^**c**^
Survival time, days	173 [169 to 179]	164 [156 to 172]	164 [156 to 173]	**0.027**^**c**^
Mortality at 2 years	34 (21)	42 (34)	37 (37)	**0.006**^**a,c**^
Survival time, days	638 [608 to 669]	565 [520 to 610]	562 [512 to 613]	**0.004**^**a,c**^
Mortality at 3 years	49 (31)	53 (46)	44 (46)	**0.015**^**a**^
Survival time, days	916 [865 to 966]	787 [715 to 859]	778 [698 to 857]	**0.006**^**a,c**^
Length of hospital stay, days	7 [4]	8 [4]	9 [6]	**0.001**^**a,d**^
NIMV	4 (2.3)	22 (18)	73 (70)	** <0.001**^**c,f**^
IMV	3 (1.7)	2 (1.6)	12 (12)	** <0.001**^**c,f**^
ICU admission	5 (2.9)	9 (7.2)	44 (43)	** <0.001**^**a,c,e**^
New ECOPD after discharge^g^	21 (12)	25 (21)	25 (25)	**0.023**^**c**^
Readmission for ECOPD^g^	4 (2.3)	2 (1.6)	3 (2.9)	0.837

Data are presented as the number of patients (percentage) or medians [interquartile range], except for survival time, which is calculated as the mean estimate [95%CI]. Percentages are calculated for non-missing data. In bold significant values.

^a^ and ^b^*p* < 0.05 and *p* < 0.001 for comparison between patients with normocapnia and hypercapnia, respectively; ^c^ and ^d^*p* < 0.05 and *p* < 0.001 for comparison between patients with normocapnia and acidosis, respectively;

^e^ and ^f^*p* < 0.05 and *p* < 0.001 for comparison between patients with hypercapnia and acidosis, respectively;

^g^ within 30 days of discharge.

*NIMV* and *IMV* indicate non-invasive and invasive mechanical ventilation, respectively, *ICU* Intensive care unit, *ECOPD* Exacerbation of the chronic obstructive pulmonary disease.

### Factors associated with mortality at 1 year

The multivariable Cox regression model (Table 3), with normocapnic patients as the reference group, showed a significant and increased mortality risk for those with hypercapnia (HR 1.91; 95%CI 1.09 to 3.35) and those with acidosis (HR 2.14; 95%CI 1.21 to 3.78). Moreover, older patients (≥80 years), overweight patients, and patients with more than two previous ECOPD episodes also had an increased risk of death at 1 year.

**Table 3:** Multivariable Cox regression model assessing factors associated with mortality at 1 year, evaluated with the three study groups

Variables	HR	95%CI	*p*-value
Study groups			
Patients with normocapnia	1	–	–
Patients with hypercapnia	1.91	1.09 to 3.35	0.023
Patients with acidosis	2.14	1.21 to 3.78	0.009
Age			
<65 years	1	–	–
65 to 79 years	1.89	1.00 to 3.59	0.051
≥80 years	3.37	1.63 to 6.97	0.001
BMI			
<18 kg/m^2^	0.85	0.26 to 2.82	0.790
18 to 24 kg/m^2^	1	–	–
25 to 29 kg/m^2^	0.48	0.26 to 0.92	0.026
≥30 kg/m^2^	0.81	0.43 to 1.51	0.497
Previous ECOPD ≥2	3.15	2.00 to 4.95	<0.001

Harrell’s C statistic is 0.71 and Somers’ D statistic is 0.42.

*HR* indicates hazard ratio, *CI* Confidence interval, *BMI* Body mass index, *ECOPD* Exacerbation of the chronic obstructive pulmonary disease.

The multivariable Cox regression model, using the threshold of 55 mmHg for PaCO_2_ (Table 4), showed that patients with PaCO_2_ ≥ 55 mmHg either with acidosis (HR 1.95; 95%CI 1.12 to 3.39) or without (HR 2.87; 95%CI 1.60 to 5.14) had an increased risk of death at 1 year with respect to those without acidosis and with PaCO_2_ < 55 mmHg. In the same model, again, older patients and those with more than two previous ECOPD, together with the presence of LTOT, also had a higher mortality risk.

**Table 4:** Multivariable Cox regression model assessing factors associated with mortality at 1 year, evaluated according to the presence of respiratory acidosis and the cut-off of 55 mmHg of PaCO_2_

Variables	HR	95%CI	*p*-value
Respiratory acidosis and hypercapnia			
Patients without acidosis (pH ≥ 7.35) and with PaCO_2_ <55 mmHg	1	–	–
Patients without acidosis (pH ≥ 7.35) and with PaCO_2_ ≥55 mmHg	2.87	1.60 to 5.14	<0.001
Patients with acidosis (pH < 7.35) and with PaCO_2_ <55 mmHg	1.97	0.69 to 5.61	0.202
Patients with acidosis (pH < 7.35) and with PaCO_2_ ≥55 mmHg	1.95	1.12 to 3.39	0.019
Age			
<65 years	1	–	–
65 to 79 years	1.67	0.88 to 3.17	0.119
≥80 years	2.96	1.44 to 6.05	0.003
Presence of LTOT	2.37	1.46 to 3.86	0.001
Previous ECOPD ≥2	2.67	1.69 to 4.23	<0.001

Harrell’s C statistic is 0.74 and Somers’ D statistic is 0.48.

*HR* indicates hazard ratio, *CI* Confidence interval, *LTOT* Long-term oxygen therapy, *ECOPD* Exacerbation of the chronic obstructive pulmonary disease.

## Discussion

In patients with COPD, the presence of hypercapnia is a marker of functional severity and progressive systemic involvement [3]. In hospitalised patients with ECOPD, hypercapnia is considered a marker of severity only if associated with acidosis [10]; this is the only selection criterion for NIMV treatment [8]. We demonstrate that hypercapnia at admission, even if not associated with acidosis, may worsen the prognosis of ECOPD patients both during hospitalisation and in the 3-year follow-up after discharge. Moreover, the threshold at admission of PaCO_2_ above 55 mmHg, rather than 45, allows better identification of patients “*at risk*” for a worse prognosis, regardless of the presence or absence of acidosis.

### May hypercapnia without acidosis be a marker of ECOPD severity?

A severe airflow limitation is a significant risk factor associated with hypercapnic failure in ECOPD patients [18], causing profound mechanical ventilatory alterations such as the presence of iPEEP and increased WOB which can be adequately addressed using NIMV [3, 8]. However, in ECOPD patients, there is solid evidence regarding the effect of the use of NIMV on the mortality rate of acidotic patients, but not in hypercapnic patients without acidosis [8], which conceptually defines these compensated patients as less severe than those with acidosis. Along these lines, the new Rome classification categorises the ECOPD hypercapnic as moderate, applying the definition of “severe ECOPD” only to acidotics [10]. Our findings challenge these considerations, and some additional clarifications are needed.

First, the limited evidence profile of NIMV efficacy in hypercapnic non-acidotic ECOPD stems from only a few studies (and patients), and these studies also present some methodological concerns [8]. Keenan’s study [19], for example, a randomised-controlled trial (RCT), considering 52 patients only and powered for a reduction of LHS and not for mortality, considered the application of bilevel NIMV for a few hours a day and not continuously for a more reasonable time. Additionally, the mean initial settings provided in that study likely resulted in low inspiratory pressures and had a less pronounced effect. Physiologically, high-intensity pressure ventilation is more effective than low-intensity in decreasing elevated PaCO_2_, reducing inspiratory effort, and alleviating dyspnoea [20]. Moreover, patients ventilated with high-intensity pressures may have significant advantages in terms of the need for endotracheal intubation (ETI) [21] and short-term mortality [22]. Finally, Keenan’s study considered patients with a relatively low mean PaCO_2_ (50 mmHg), indicating mild to moderate ventilatory impairment, which may show slight improvement with NIMV during hospitalisation.

Second, the evaluation of the efficacy of NIMV on hypercapnic ECOPD has been primarily assessed by mortality outcomes [8], while more solid findings may be obtained by considering ETI [8, 23]. In fact, a prospective multicentre RCT conducted in 19 Chinese hospitals recorded that, in a subgroup of ECOPD ventilated patients (*N* = 151) who were not acidotic, there was a reduction in reporting meeting criteria for ETI (3% vs 11%), similar to patients with acidosis (7% vs 27%) [23].

Third, in the context of ECOPD, acidosis is an intercurrent phenomenon related to individual and temporal factors of metabolic compensation, primarily achieved through renal retention of bicarbonate [24], and therefore not closely associated with the severity of ventilatory lung failure. Patients with acute kidney injury (AKI), a common complication occurring in 21% of ECOPD [25] impairing the kidney’s capacity to compensate for acidosis, exhibit several worse outcomes with respect to patients without AKI,, including higher mortality rates at 30 days (14% vs 4%), ETI (54% vs 14%), and more severe hypercapnic acidosis [26]. Conversely, the development of sufficient metabolic compensation and adequate renal function significantly decreases mortality [27]. The measure of hypercapnia, in contrast to acidosis, reflects a mechanical lung alteration only, without any metabolic interference.

Fourth, a retrospective analysis evaluating the outcomes of 347 hospitalised patients with ECOPD and categorised according to the Rome classification, found a higher mortality risk after 3 years pf follow-up when patients presented a severe grade (acidosis) or moderate grade (hypercapnia only) than those with mild grade (no hypercapnia). The study highlighted the prognostic role of hypercapnia, regardless of acidosis [11].

Finally, in a real-life scenario, very recent evidence indicates that the use of NIMV for ECOPD in patients with hypercapnia achieves an overall rate of success (need for ETI and/or all-cause in-hospital death) similar to that of patients with acidosis (23% vs 20%) [28]. The evidence about the role of the compensated hypercapnic patients should be updated.

### What role may hypercapnia play in the chronicity of the disease?

In COPD patients, the presence of hypercapnia may be persistent, also after ECOPD [3]. A long-term study of patients with stable COPD showed shorter survival in patients with chronic hypercapnia than in those with normocapnia (5 vs 6.5 years) [29]; of note, measures of severe lung impairment, related to alveolar hypoventilation, such as PaCO_2_ (HR = 1.03; 95%CI 1.01 to 1.04) and of airflow limitation such as FEV_1_% of predicted (HR = 0.98; 95%CI 0.97 to 0.99), together with age (HR = 1.04; 95%CI 1.01 to 1.08) were considered independent prognostic risk factors [29]. Similarly, in our study the presence of hypercapnia may represent a sign of a more severe COPD, as confirmed by the significant differences in terms of airflow obstruction (FEV_1_/FVC), severity of airflow limitation (FEV_1_% predicted), and in the prevalence of patients requiring LTOT (Table 1). Therefore, a more severe COPD may have a worse prognosis. We report a higher mortality rate for hypercapnic patients, up to 3 years (Table 2), with hypercapnia or acidosis both increasing the mortality risk at 1 year (Table 3); interestingly, in our model, older age was a negative prognostic factor [29].

Although in a smaller cohort, a pilot study considering Asian ECOPD patients found a similar risk between hypercapnic and acidotic patients at admission in terms of life-threatening events and mortality risk [30]. However, in the regression model in that study PaCO_2_ was included as a continuous measure, rather than using a specific threshold (55 mmHg), which helps identify patients “*at risk*” for a hypothetical treatment, regardless of acidosis. Interestingly, in our regression model (Table 4), the presence of acidosis, if associated with a relatively small increase of PaCO_2_ (<55 mmHg), was not a prognosis risk factor (HR 1.97; 95%CI 0.69 to 5.61), suggesting again the greater role of the severe and chronic ventilatory impairment than of the intercurrent metabolic acidosis.

### Can we therefore change the prognosis of hypercapnic patients, even if they do not have acidosis?

The domiciliary use of NIMV in hypercapnic COPD has been demonstrated in a recent meta-analysis to be associated with a lower risk of mortality than all-cause hospital admission, and lower need for ETI [31]. Although a prospective Dutch study reported that the improvement in hypercapnia raised survival in chronically ventilated patients [32], other reports indicate that the survival effect may be particularly evident when NIMV is targeted at reducing PaCO_2_ levels, indicating the effect of different ventilatory parameters on the efficacy of this treatment in COPD patients [33–35]. Moreover, a recent meta-analysis involving 1482 patients confirmed that the chronic use of NIMV significantly reduces mortality, especially in a subgroup analysis of COPD patients with higher baseline levels of PaCO_2_ (>55 mmHg)[36]. Similarly, our regression model (Table 4) reports a significant mortality risk in patients with a level of PaCO_2_ greater than 55 mmHg.

Significant differences in the change in PaCO_2_ were found in patients ventilated with inspiratory pressure levels of at least 18 cm H_2_O, in patients who used a ventilator for at least 5 h per night, and in patients with a baseline PaCO_2_ level of at least 55 mmHg[37]. Similar studies have confirmed the importance of setting ventilatory pressures at a high level, thereby improving arterial blood gas levels [34, 38]. To enhance the efficacy and tolerability of NIMV, it may be beneficial to provide strategies for optimising treatment.

### Strengths and limitations

Major strengths of the study include the originality of our approach, the selection criteria used for enrolling patients, and the potential to propose a threshold for PaCO_2_, which helps identify patients with a worse prognosis. As for the limitations, we should mention the missing data on patients using domiciliary NIMV, which means that we cannot rule out the possibility that some hypercapnic patients at admission may have been chronically hypercapnic. However, this aspect may also be present in patients with acidosis and would then be diluted between the two study groups. Moreover, the prevalence of patients with LTOT as a sign of disease severity and a proxy for chronic need for NIMV may help address this issue, and appears similar between hypercapnia and acidosis.

In conclusion, we demonstrate that in hospitalised patients with ECOPD, hypercapnia at admission may be associated with a worse outcome regardless of the presence of acidosis. Considering a threshold of PaCO_2_ of 55 mmHg may help identify patients who have a worse prognosis and, therefore, are potentially eligible for NIMV in the acute setting, thereby expanding the indication of ERS guidelines and leading the way for the design of a specific study to demonstrate its efficacy. In this context, the severity and chronicity of ventilatory impairment might be more relevant than an intercurrent acidosis.

## Supplementary Information

Below is the link to the electronic supplementary material.

Supplementary file1 (DOCX 54 KB)

Supplementary file2 (JPG 100 KB)

Supplementary file3 (JPG 330 KB)

## Data Availability

The datasets used and/or analysed during the current study are available from the corresponding author on reasonable request.
