# Supplementary material for: Hypercapnia at admission, regardless of acidosis, may worsen the outcome of hospitalised patients with chronic obstructive pulmonary disease exacerbations
Source: Intern Emerg Med. 2026 Feb 9;21(4):1203–13. doi: 10.1007/s11739-026-04279-0 (PMC13263262; doi:10.1007/s11739-026-04279-0)
Supplement: Supplementary file 1 — Supplementary file1 (DOCX 54 KB) [file 11739_2026_4279_MOESM1_ESM.docx]

**SUPPLEMENTARY TABLES**

**Supplementary Table 1.** Clinical and laboratory variables.

| Variables | Total cohort  (N=407) | Patients with normocapnia  (N=176) | Patients with hypercapnia  (N=126) | Patients with acidosis  (N=105) | p-value |
| --- | --- | --- | --- | --- | --- |
| At admission |  |  |  |  |  |
| Respiratory rate, b/min | 24 [8] | 24 [6] | 24 [8] | 24 [6] | 0.192 |
| Heart rate, b/min | 94 [24] | 92 [22] | 97 [22] | 94.5 [29.5] | 0.279 |
| pH | 7.39 [0.9] | 7.44 [0.05] | 7.39 [0.04] | 7.3 [0.07] | **<0.001^b,d,f^** |
| PaCO_2_, mmHg | 48.1 [21.2] | 37.8 [5.8] | 52.5 [11] | 66.6 [17.5] | **<0.001^b,d,f^** |
| PaO_2_/FiO_2_ | 259.6 [101.4] | 281.9 [95] | 246.5 [97.1] | 234.8 [125.2] | **<0.001^b,d^** |
| HCO_3_^-^, mmol/L | 28 [7.7] | 24.8 [3.8] | 32.1 [7.1] | 30.8 [7] | **<0.001^b,d^** |
| BE, mmol/L | 2.4 [5.8] | 0.9 [3.5] | 6 [5.8] | 2.6 [6.2] | **<0.001^b,d,f^** |
| Leucocytes, 10^9^/L | 10.3 [5.9] | 10.3 [5.6] | 9.5 [5] | 11.2 [6.6] | 0.301 |
| Haemoglobin, g/L | 139 [27] | 138 [26] | 136.5 [23] | 144 [32] | 0.139 |
| CRP, mg/dL | 3.9 [8.8] | 3.8 [9.2] | 4.6 [8.5] | 2.5 [7] | **0.046^e^** |
| Creatinine, mg/dL | 0.9 [0.3] | 0.9 [0.3] | 0.9 [0.3] | 1 [0.4] | **0.009^a,e^** |
| At day 3 |  |  |  |  |  |
| pH | 7.41 [0.06] | 7.43 [0.04] | 7.4 [0.04] | 7.4 [0.07] | **0.013^a,c^** |
| PaCO_2_, mmHg | 49.2 [12.4] | 42.9 [7.9] | 52.7 [6] | 50.8 [15] | **<0.001^b,d^** |
| PaO_2_/FiO_2_ | 281 [59] | 292.9 [48.1] | 275.7 [54.2] | 276.5 [79.2] | 0.177 |
| HCO_3_^-^, mmol/L | 30.3 [7] | 27.2 [4.4] | 32.3 [5.3] | 31.1 [7.4] | **<0.001^b,c^** |
| BE, mmol/L | 4.5 [4.5] | 3 [3.6] | 6 [4.4] | 5.1 [5.6] | **<0.001^b,c^** |
| Leucocytes, 10^9^/L | 10.7 [4.8] | 10.9 [5.1] | 10.4 [4.5] | 10.7 [4.6] | 0.651 |
| Haemoglobin, g/L | 130 [24] | 130 [24.5] | 128.5 [26.5] | 131 [26] | 0.757 |
| CRP, mg/dL | 1.3 [3] | 1.7 [3.4] | 1 [2.7] | 1.1 [2.5] | 0.108 |
| Creatinine, mg/dL | 0.9 [0.4] | 0.9 [0.5] | 0.8 [0.4] | 0.8 [0.4] | 0.185 |
| Treatments |  |  |  |  |  |
| Systemic corticosteroids | 373 (93) | 155 (90) | 117 (96) | 101 (96) | **0.039** |
| Antibiotics | 339 (87) | 147 (87) | 100 (84) | 92 (88) | 0.575 |
| Antibiotics used ≥2^g^ | 82 (21) | 34 (21) | 26 (22) | 22 (22) | 0.858 |
| Duration of antibiotics, days | 7 [4.5] | 7 [5] | 7 [4.5] | 7 [3] | 0.715 |

Data are presented as the number of patients (percentage) or medians [interquartile range]. Percentages are calculated for non-missing data. In bold significant values.

^a^ and ^b^ p<0.05 and p<0.001 for comparison between patients with normocapnia and hypercapnia, respectively;

^c^ and ^d^ p<0.05 and p<0.001 for comparison between patients with normocapnia and acidosis, respectively;

^e^ and ^f^ p<0.05 and p<0.001 for comparison between patients with hypercapnia and acidosis, respectively;

^g^ occurring in the preceding year.

The percentage of antibiotics used was as follows: fluoroquinolones (52%), penicillins (17%), cephalosporins (4.2%), macrolides (1.2%) and carbapenems (0.9%). There were no differences according to study groups.

Abbreviations*:* PaCO_2_, partial pressure of arterial carbon dioxide; PaO_2_/FiO_2_, the ratio of partial pressure of arterial oxygen to the fraction of inspired oxygen; HCO_3_^−^, serum bicarbonate; BE, base excess; CRP, C-reactive protein.

**Supplementary Table 2.** Microbiological variables.

| Variables | Total cohort  (N=407) | Patients with normocapnia  (N=176) | Patients with hypercapnia  (N=126) | Patients with acidosis  (N=105) | p-value |
| --- | --- | --- | --- | --- | --- |
| Patients with positive cultures^a^ | 76 (19) | 34 (19) | 25 (20) | 17 (16) | 0.745 |
| Aspergillus spp. | 2 (2.7) | 1 (2.9) | 1 (4.2) | 0 (0) | >0.999 |
| Candida spp. | 2 (2.7) | 0 (0) | 1 (4.2) | 1 (5.9) | 0.295 |
| Corynebacterium spp. | 2 (2.7) | 1 (2.9) | 1 (4.2) | 0 (0) | >0.999 |
| Haemophilus spp. | 14 (19) | 5 (15) | 3 (12) | 6 (35) | 0.157 |
| Klebsiella spp. | 1 (1.3) | 0 (0) | 1 (4.2) | 0 (0) | - |
| Moraxella spp. | 4 (5.3) | 2 (5.9) | 1 (4.2) | 1 (5.9) | >0.999 |
| Mycobacterium no-tuberculosis | 1 (1.3) | 1 (2.9) | 0 (0) | 0 (0) | >0.999 |
| Pseudomonas aeruginosa | 20 (27) | 6 (18) | 11 (46) | 3 (18) | **0.049** |
| Serratia spp. | 1 (1.3) | 0 (0) | 0 (0) | 1 (5.9) | 0.227 |
| Staphylococcus spp. | 6 (8) | 1 (2.9) | 4 (17) | 1 (5.9) | 0.157 |
| Stenotrophomonas maltophilia | 1 (1.3) | 1 (2.9) | 0 (0) | 0 (0) | - |
| Streptococcus pneumoniae | 13 (17) | 11 (32) | 0 (0) | 2 (12) | **0.002^b^** |
| Polymicrobial aetiology | 7 (9.3) | 4 (12) | 1 (4.2) | 2 (12) | 0.596 |
| Patients with virus positivity^c^ | 32 (14) | 15 (20) | 12 (21) | 5 (11) | 0.378 |
| Influenza B virus | 3 (9.4) | 2 (13) | 0 (0) | 1 (20) | 0.365 |
| Respiratory syncytial virus | 5 (16) | 2 (13) | 3 (25) | 0 (0) | 0.543 |
| Rhinovirus | 5 (16) | 2 (13) | 3 (25) | 0 (0) | 0.543 |
| Parainfluenza virus type 1 | 3 (9.4) | 3 (20) | 0 (0) | 0 (0) | 0.259 |
| Parainfluenza virus type 3 | 2 (6.3) | 2 (13) | 0 (0) | 0 (0) | 0.637 |
| Parainfluenza virus type 4 | 1 (3.1) | 1 (6.7) | 0 (0) | 0 (0) | >0.999 |

Data are presented as the number of patients (percentage). Percentages are calculated for non-missing data. In bold significant values.

^a^ Percentages are calculated on total patients with positive cultures in sputum in each group (34, 24 and 17 in patients with normocapnia, hypercapnia, and acidosis, respectively).

^b^ p<0.05 for comparison between patients with normocapnia and hypercapnia;

^c^ Percentages are calculated on total patients with virus positivity (15, 12 and 5 in patients with normocapnia, hypercapnia, and acidosis, respectively).

**LEGENDS TO SUPPLEMENTARY FIGURES**

**Supplementary Figure 1.** Study flow diagram.

Abbreviations: ECOPD, exacerbation of the chronic obstructive pulmonary disease.

**Supplementary Figure 2**. ROC curve used to identify the cut-off of PaCO_2_ that better identifies the mortality at 1 year.

Abbreviations: ROC, receiver operating characteristic; AUC, area under the curve; SE, standard error; CI, confidence interval.
