# Supplementary figures and images for: Hypercapnia at admission, regardless of acidosis, may worsen the outcome of hospitalised patients with chronic obstructive pulmonary disease exacerbations
Source: Intern Emerg Med. 2026 Feb 9;21(4):1203–13. doi: 10.1007/s11739-026-04279-0 (PMC13263262; doi:10.1007/s11739-026-04279-0)

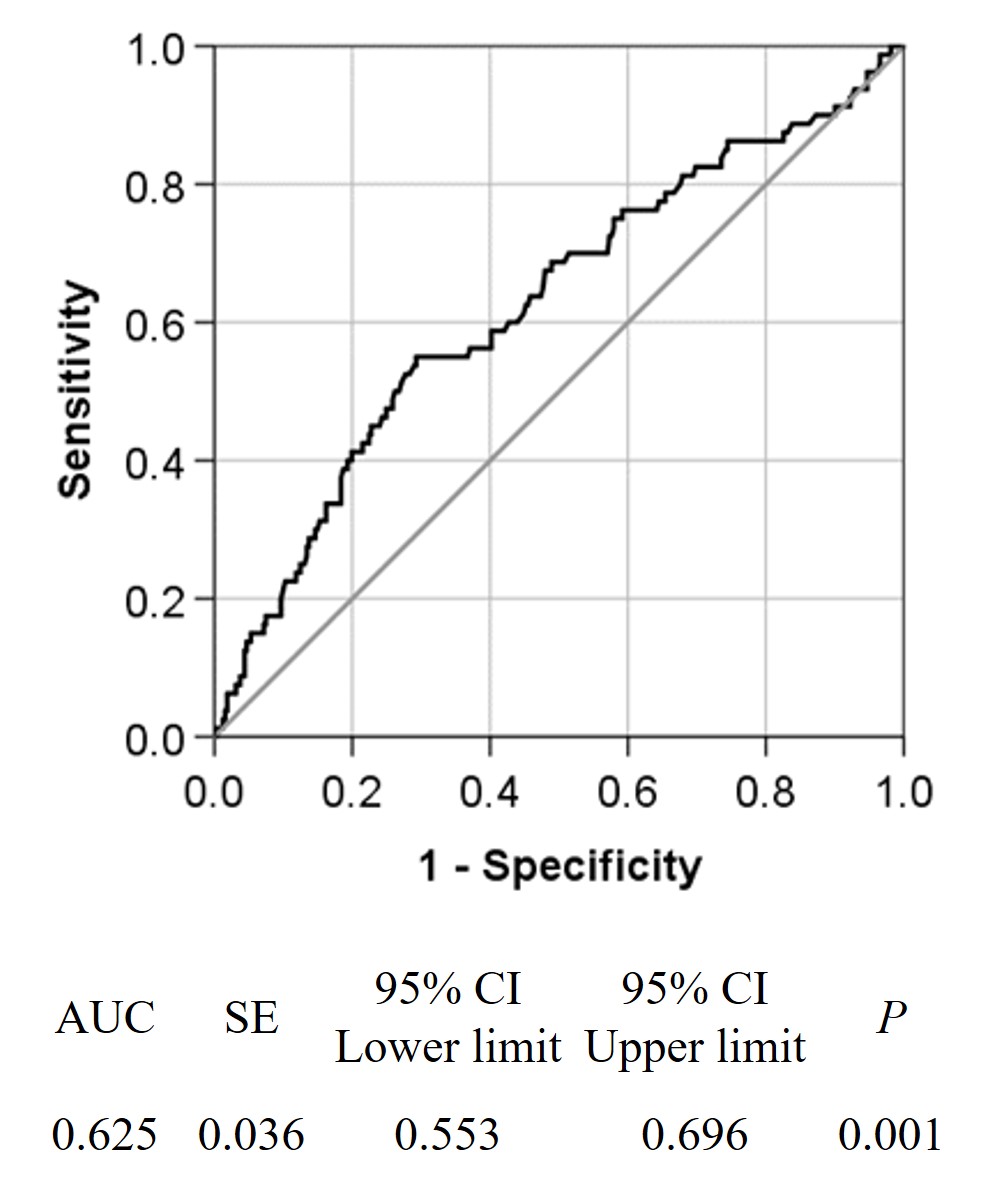

Supplement: Supplementary file 2 — Supplementary file2 (JPG 100 KB) [file 11739_2026_4279_MOESM2_ESM.jpg]

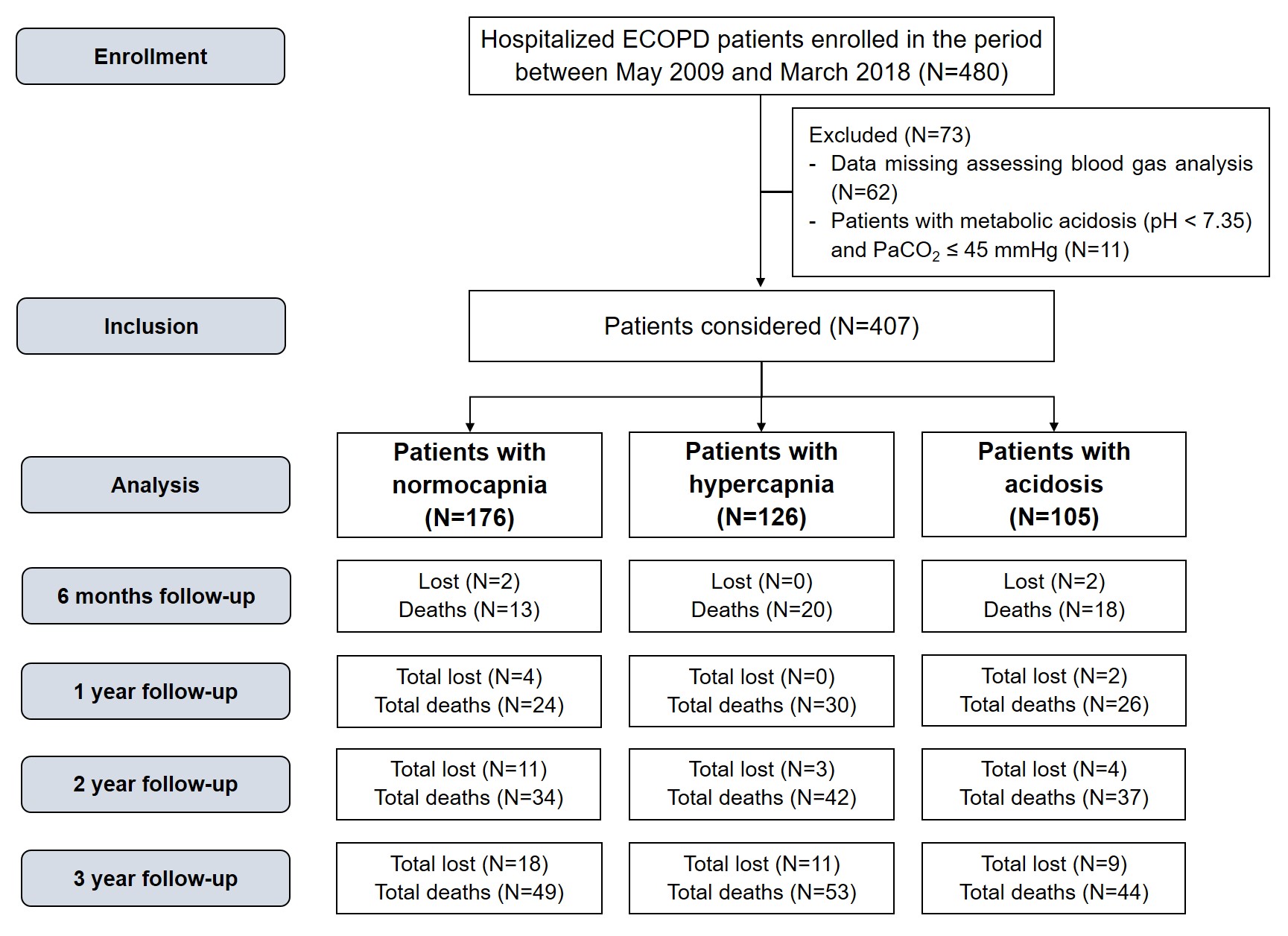

Supplement: Supplementary file 3 — Supplementary file3 (JPG 330 KB) [file 11739_2026_4279_MOESM3_ESM.jpg]
